# Inducible IFN-γ Expression for MHC-I Upregulation in Devil Facial Tumor Cells

**DOI:** 10.3389/fimmu.2018.03117

**Published:** 2019-01-14

**Authors:** Chrissie E. B. Ong, Alan Bruce Lyons, Gregory M. Woods, Andrew S. Flies

**Affiliations:** ^1^Menzies Institute for Medical Research, College of Health and Medicine, University of Tasmania, Hobart, TAS, Australia; ^2^School of Medicine, College of Health and Medicine, University of Tasmania, Hobart, TAS, Australia

**Keywords:** transmissible tumor, DFTD, IFN-γ, MHC-I, Tet-Off system, inducible, PD-L1, apoptosis

## Abstract

The Tasmanian devil facial tumor (DFT) disease has led to an 80% reduction in the wild Tasmanian devil (*Sarcophilus harrisii*) population since 1996. The limited genetic diversity of wild devils and the lack of MHC-I expression on DFT cells have been implicated in the lack of immunity against the original DFT clonal cell line (DFT1). Recently, a second transmissible tumor of independent origin (DFT2) was discovered. Surprisingly, DFT2 cells do express MHC-I, but DFT2 cells appear to be on a trajectory for reduced MHC-I expression *in vivo*. Thus, much of the ongoing vaccine-development efforts and conservation plans have focused on MHC-I. A major limitation in conservation efforts is the lack of species-specific tools to understand Tasmanian devil gene function and immunology. To help fill this gap, we developed an all-in-one Tet-Off vector system to regulate expression of IFN-γ in DFT cells (DFT1.Tet/IFN-γ). IFN-γ can have negative effects on cell proliferation and viability; thus, doxycycline was used to suppress IFN-γ production whilst DFT1.Tet/IFN-γ cells were expanded in cell culture. Induction of IFN-γ following removal of doxycycline led to upregulation of MHC-I but also the inhibitory checkpoint molecule PD-L1. Additionally, DFT1.Tet/IFN-γ cells were capable of stimulating MHC-I upregulation on bystander wild type DFT cells in co-culture assays *in vitro*. This system represents a major step forward in DFT disease immunotherapy and vaccine development efforts, and ability to understand gene function in devils. Importantly, the techniques are readily transferable for testing gene function in DFT2 cells and other non-traditional species.

## Introduction

The Tasmanian devil facial tumor disease (DFTD) is caused by clonal transmissible cancers (1, 2). The devil facial tumor 1 (DFT1) is primarily responsible for an 80% reduction in the wild Tasmanian devil (*Sarcophilus harrisii*) population (3). The recently discovered devil facial tumor 2 (DFT2) is a second transmissible tumor of independent origin that introduces additional uncertainty about the long-term persistence of wild devils (2). Despite genetic mismatches between tumor and host, most prominently in major histocompatibility complex (MHC) alleles, evidence of anti-tumor immune responses are rare (4, 5). The limited evidence of host immune response against DFT suggests effective immune evasion by the tumor cells. One such mechanism is the epigenetic loss of MHC class I (MHC-I) molecules from the surface of DFT cells (6), which facilitates escape from cytotoxic T cell recognition.

Several preventative and therapeutic approaches have been pursued to manage the spread of this debilitating disease, with vaccination and immunotherapy having better success than chemotherapy (7). Vaccines for DFTD have been formulated using killed DFT cells, either frozen/thawed, sonicated, or irradiated for safety reasons. The first immunization trial using killed DFT cells with the adjuvant Montanide failed to trigger any humoral or cytotoxic response (8) but the following trial, with the addition of CpG, elicited antibody production and cytotoxicity in five out of six immunized devils (9). The vaccine however, was not fully protective as tumors developed upon challenge with live DFT cells.

Treatment of DFT cells with recombinant devil IFN-γ induces the expression of MHC-I heavy chain and genes essential for MHC-I antigen processing and presentation including β_2_-microglobulin (β_2_-m) (6, 10). A vaccine consisting of DFT cells pre-treated with recombinant IFN-γ in cell culture and then killed and mixed with adjuvants was used to prime the devil immune system against DFT cells (11, 12). Serum antibodies against MHC-I^+^ and MHC-I^−^ DFT cells were detected but the immunized devils developed tumors following infection with live DFT cells (11). The devils were given immunotherapy comprising of live IFN-γ treated MHC-I^+^ DFT cells and tumor regression was induced in three of the six immunized devils (11). Although prior exposure to DFT vaccines was necessary to initiate tumor regression, this observation substantiates the prospect of developing a live tumor cell vaccine for DFTD. A live-attenuated DFTD vaccine could be more effective than killed-cell vaccine preparations, as has been the case for conventional live-attenuated vaccines that mimic a natural infection (13–17).

We have previously shown that IFN-γ also upregulates the inhibitory checkpoint molecule programmed death ligand 1 (PD-L1) on DFT1 and DFT2 cells (18). Binding of PD-L1 to PD-1 blocks activation signals, particularly co-stimulatory signals associated with antigen receptors on T cells and B cells (19–21). This occurs via immunoreceptor tyrosine-based switch motifs (ITSMs) and immunoreceptor tyrosine-based inhibitory motifs (ITIMs) that recruit phosphatases that dephosphorylate downstream signal transduction pathways (19). Upregulation of PD-L1 on DFT cells appears to lag behind MHC-I upregulation and peak expression levels of PD-L1 are at least an order of magnitude lower than β_2_-m. This could provide a window of opportunity for anti-DFT responses and immune checkpoint immunotherapy that blocks the PD-1/PD-L1 pathway.

In developing a live-attenuated MHC-I^+^ DFT cell vaccine, an alternative to treating DFT cells with exogenous IFN-γ is to generate a DFT cell line that expresses IFN-γ. This would be more cost-effective than using purified IFN-γ and has the potential to be used as an immunotherapy to induce MHC-I upregulation on bystander MHC-I^−^ DFT cells in diseased devils. One complication with using IFN-γ for MHC-I upregulation is the anti-proliferative and pro-apoptotic properties of IFN-γ (22–27) that will hinder the development of IFNγ-expressing DFT cells. Prolonged DFT cell culture with IFN-γ, even at doses as low as 5 ng/ml, results in reduced proliferation and viability (28). The large quantity of cells and secreted IFN-γ needed for a live-attenuated vaccine to be effective *in vivo* would be difficult to obtain due to the effects of IFN-γ *in vitro*. On the contrary, these anti-proliferative and pro-apoptotic effects of IFN-γ would be desirable aspects of a live-attenuated vaccine.

We hypothesized that the Tet-Off system, a tetracycline (tet)-controlled regulatory system (29) could be employed to regulate IFN-γ expression in DFT cells. The Tet-Off system regulates gene expression by using a tetracycline (tet)-controlled transactivator (tTA), which is active in the absence of tetracycline, to initiate transcription of genes downstream to a tetracycline response element and minimal CMV-promoter enhanced (TCE) promoter (30). In the presence of tetracycline and its derivatives (e.g., doxycycline), tTA is bound by tetracycline and loses its DNA binding ability, blocking transcription of genes reliant on the TCE.

In this study, we demonstrate tight regulation of functional IFN-γ production by DFT cells (referred to as DFT1.Tet/IFN-γ) using doxycycline to switch IFN-γ on/off. MHC-I and inhibitory checkpoint molecule PD-L1 were upregulated in response to IFN-γ induction. Meanwhile, the anti-proliferative and pro-apoptotic effects of IFN-γ were prevented by inhibiting IFN-γ expression using doxycycline. Importantly, we also observed an *in vitro* paracrine signaling bystander effect that upregulated MHC-I on wild type DFT cells co-cultured with DFT1.Tet/IFN-γ cells. This system can be applied to other genes to allow understanding of gene function in Tasmanian devils and opens pathways to develop live-attenuated vaccines and/or immunotherapies for DFTD.

## Materials and Methods

### Plasmid Construction

**Figure 1:**
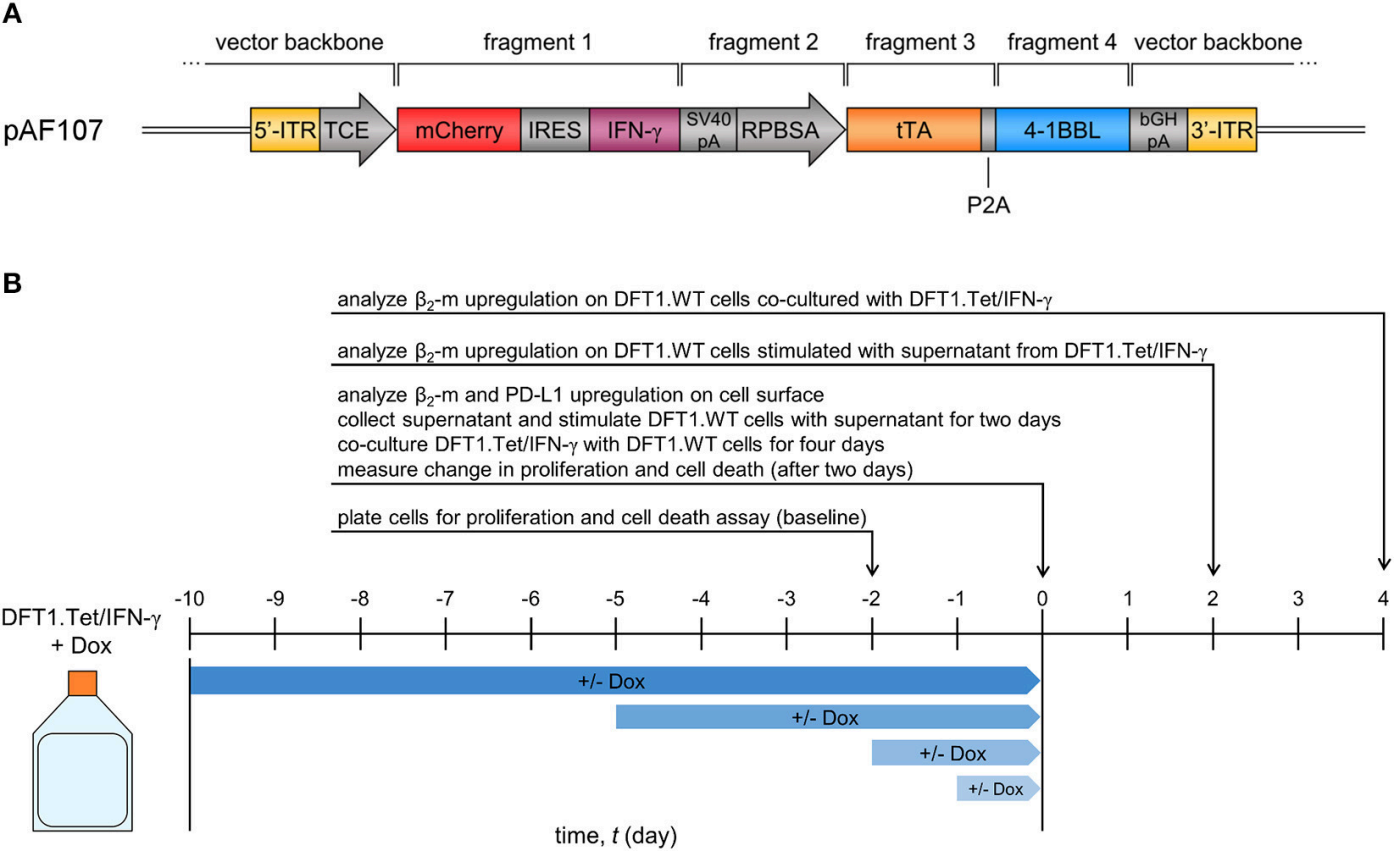
Vector and study design of Tet-inducible IFN-γ-expressing DFT1 cells (DFT1.Tet/IFN-γ). **(A)** Expression vector for tetracycline (tet)-controlled inducible IFN-γ expression in DFT1 cells. *ITR*, inverted terminal repeats; *TCE*, tet-responsive promoter; *IRES*, internal ribosomal entry site; *tTA*, tet-controlled transactivator. **(B)** Timeline for dox+/– treatments of DFT1.Tet/IFN-γ used in subsequent experiments. DFT1.Tet/IFN-γ cultured in 100 ng/ml doxycycline (Dox) was split into four groups with doxycycline removed or doxycycline continually replenished for 1, 2, 5, or 10 days. Cells were analyzed for: (i) surface β_2_-m and PD-L1 upregulation; (ii) ability to stimulate MHC-I upregulation on wild type DFT1 cells (DFT1.WT) using the supernatant or by co-culture; and (iii) cell proliferation and viability in response to doxycycline removal.

An expression vector for Tet-Off inducible devil IFN-γ (referred to as pAF107) was constructed by fusing multiple fragments using PCR and cloning methods (Figure 1A). The vector backbone for pAF107 was generated from Sleeping Beauty (SB) transposon plasmid pSBtet-RH (Addgene, Cambridge, MA, USA), linearized by PCR. pSBtet-RH was a gift from Eric Kowarz (30). The inserts for pAF107 consisted of four fragments that were prepared as follows. Fragment 1 is a cassette containing red fluorescent protein mCherry, followed by an internal ribosome entry site (IRES), and devil IFN-γ cDNA (mCherry-IRES-IFNγ). This cassette was obtained from plasmid pAF67, which was constructed by cloning devil IFN-γ cDNA [PCR-amplified from a pre-existing plasmid, pAF23 (18)] into the multiple cloning site (MCS) of PCR-linearized pTRE-Dual2 (Clontech Laboratories, Mountain View, CA, USA). Fragment 2 (SV40pA-RPBSA) was obtained from pSBtet-RH, and fragment 3 (tTA) was obtained from pTet-DualOFF (Clontech). Fragment 4 (P2A-devil 41BBL) was obtained from a pre-existing plasmid encoding devil 4-1BBL, pAF56.1. All fragments were obtained by PCR with overlapping ends using KAPA Hotstart HiFi Master Mix (Kapa Biosystems, Wilmington, MA, USA) (see Supplementary Table 1 for primers and PCR cycling conditions). The fragments were fused together by overlap extension PCR prior to cloning into pAF107 vector backbone using NEBuilder® HiFi DNA Assembly Cloning Kit (NEB). All assembled plasmids were transformed into NEB® 5-alpha competent *Escherichia coli* (NEB) following manufacturer's instructions. Plasmid integrity was confirmed by Sanger sequencing using BigDye® Terminator v3.1 Cycle Sequencing Kit (Applied Biosystems (ABI), Foster City, CA, USA) and analysis on 3500xL Genetic Analyzer (ABI).

### Cell Culture

The devil facial tumor 1 (DFT1) cell line C5065 (strain 3), provided by A-M Pearse and K. Swift of the Department of Primary Industries, Parks, Water and Environment (DPIPWE) (Hobart, Australia) was used for all experiments. Cells were cultured in complete RPMI medium, RPMI 1640 medium with L-glutamine (Gibco, Waltham, MA, USA), supplemented with 10% heat inactivated fetal bovine serum (Bovogen Biologicals, Melbourne, Australia), 1% Antibiotic-Antimycotic (Gibco), 10 mM HEPES (Gibco), and 0.1 mM 2-mercaptoethanol (Sigma-Aldrich, St. Louis, MO, USA) at 35°C with 5% CO_2_. Tet-Off inducible IFNγ-expressing DFT cells (DFT1.Tet/IFN-γ) were cultured in complete RPMI medium with 100 ng/ml doxycycline (Clontech) unless indicated otherwise.

### Transfection

C5065 cells were seeded onto a 6-well plate at a confluency between 40 and 80% and were incubated overnight. The cells were co-transfected with pAF107 and pCMV(CAT)T7-SB100 (Addgene) at a ratio of 9:1 (in μg), respectively, using a total of 1–2 μg plasmid DNA. pCMV(CAT)T7-SB100 was a gift from Zsuzsanna Izsvak (31) and is used to facilitate integration of SB transposon vectors into the genome. Plasmid DNA was diluted in serum-free RPMI and incubated for 2–5 min at room temperature. Polyethyleminine (PEI) (1 mg/ml, linear, 25 kDa; Polysciences, Warrington, PA, USA) was added to the diluted DNA at a 3:1 ratio and was mixed briefly by vortexing. The solution was incubated for 15–30 min at room temperature before adding it to the cells. The cells were incubated with the solution for 4 h, after which it was replaced with complete RPMI medium.

### Gene Induction and Suppression

For gene suppression, doxycycline (100 ng/ml) was added to the culture medium and replenished every 2 days. For gene induction, the medium was decanted, and the cells were rinsed once with PBS (Oxoid, Hampshire, UK) while remaining adhered to the surface. The cells were then harvested in PBS and pelleted at 200 × *g* for 5 min at 20°C. The cells were resuspended and cultured in complete RPMI medium in the absence of doxycycline.

### Flow Cytometric Cell Sorting

Doxycycline was removed from the culture medium at least 2 days prior to cell-sorting to turn on expression of reporter mCherry, which is co-expressed with IFN-γ under the control of inducible TCE promoter. Cells were harvested at 200 × *g* for 5 min at 20°C and resuspended in complete RPMI medium to generate a single-cell suspension. mCherry^+^ cells were selected and enriched by bulk-sorting using cell sorter Moflo Astrios EQ (Beckman Coulter). After sorting, the cells were cultured with doxycycline (100 ng/ml) and expanded for a month before undergoing a second round of enrichment by bulk-sorting.

### Detection of IFN-γ, β_2_-m, and PD-L1 mRNA by RT-PCR

Total RNA was extracted from cells using Nucleospin® RNA plus (Macherey Nagel, Bethlehem, PA, USA). RNA integrity was validated by running on a 1% agarose gel at 100 V for 30 min before proceeding to cDNA synthesis. One μg of RNA was reverse-transcribed to cDNA using GoScript^TM^ Reverse Transcription System (Promega, Madison, WI, USA). A no-reverse transcriptase (no-RT) control was included for each RNA sample to verify absence of genomic DNA contamination. IFN-γ, β_2_-m, and PD-L1 cDNA were amplified by PCR, generating products of 310, 301, and 280 bp, respectively (see Supplementary Table 2 for primers and PCR cycling conditions). The housekeeping gene GAPDH was used as a reference gene. Primers for IFN-γ, PD-L1, and GAPDH were designed using SnapGene® against mRNA sequences from the Tasmanian devil Reference Genome Devil_ref v7.0 assembly GCF_000189315.1. Primers for β_2_-m were designed as previously described (6). PCR reactions were carried out using Q5® Hot Start High-Fidelity 2X Master Mix (NEB), and the products were run on a 1% agarose gel at 100 V for 30 min.

### Analysis of MHC-I and PD-L1 Surface Expression by Flow Cytometry

Cells (1 × 10^5^ per well) were harvested in a round-bottom 96-well plate at 500 × *g* for 3 min at 4°C. The cells were blocked with 1% normal goat serum (Thermo Fisher Scientific, Waltham, MA, USA) in FACS buffer (PBS with 0.5% BSA, 0.05% NaN_3_) for 10 min on ice, followed by incubation with 0.4 μl/sample of anti-devil β_2_-m mouse antibody (gift from Hannah Siddle) (10) for 15 min on ice. After incubation, the cells were washed by adding 150 μl FACS buffer and centrifuging at 500 × *g* for 3 min at 4°C. 0.4 μg/sample of secondary antibody goat anti-mouse IgG-Alexa Fluor 488 (Thermo Fisher Scientific) was added to the cells and incubated for 15 min on ice. The cells were washed twice with FACS buffer to remove excess secondary antibody, and then incubated with mouse anti-devil PD-L1 clone 1F8 antibody (18) labeled with DyLight 650 using DyLight™ 650 Microscale Antibody Labeling Kit (Thermo Fisher Scientific) for 15 min on ice. The cells had a final rinse with FACS buffer and were resuspended in 200 μl DAPI (200 ng/ml) (Sigma-Aldrich). The cells were analyzed on Moflo Astrios EQ for β_2_-m and PD-L1 surface expression.

### Stimulation of MHC-I on C5065 Cells Using Supernatant From DFT1.Tet/IFN-γ

DFT1.Tet/IFN-γ (2 × 10^6^ cells per flask) were seeded in 25 cm^2^ cell culture flasks. After culturing for 24 h, the supernatant was collected, centrifuged at 3200 × *g* for 10 min at 4°C, and passed through a 0.22 μm filter membrane. One ml of supernatant was added to wild type C5065 cells (2 × 10^5^ cells per well) seeded on 12-well plates the day before. C5065 cells were stimulated with the supernatant for 48 h and MHC-I upregulation was quantified by flow cytometry based on β_2_-m expression (according to the method described above excluding anti-PD-L1 antibody staining). The cells were analyzed on BD FACSCanto^TM^ II (BD Biosciences, Franklin Lakes, NJ, USA).

### Stimulation of MHC-I on C5065 Cells Through Co-culture With DFT1.Tet/IFN-γ

DFT1.Tet/IFN-γ was co-cultured in triplicate at a ratio of 1:1 with C5065 cells labeled with CellTrace Far Red (Thermo Fisher Scientific) in 12-well plates (total of 1 × 10^5^ cells per well) for 4 days. The cells were analyzed for MHC-I upregulation by flow cytometry (according to the method described above) using Moflo Astrios EQ.

### Cell Proliferation Assay

Cell proliferation was analyzed by water-soluble tetrazolium-8 (WST-8) proliferation assay using Cell Counting Kit-8 (CCK-8) (Sigma Aldrich). Cells (1 × 10^4^ per well in 100 μl suspension) were inoculated in triplicate in 96-well plates. Ten microliter CCK-8 solution was added to each well and the plate was incubated for 4 h at 35°C. Absorbance at 450 nm was measured on the day of inoculation to determine the baseline absorbance of cells for each sample, and after 2 days of incubation to assess change in absorbance using plate reader Spark® 20M (Tecan, Männedorf, Switzerland).

### Cell Death Assay

Cells (2 × 10^5^ per well) were seeded in 12-well plates in triplicate. The percentage of cell death over 2 days was quantified by staining cells with DAPI (200 ng/ml) and analysis by flow cytometry using Moflo Astrios EQ.

## Results

### Regulation of IFN-γ by Doxycycline

Before developing a Tet-Off inducible expression system for IFN-γ, we conducted a preliminary experiment investigating the utility of this system in DFT cells. We transfected C5065 cells (a DFT1 cell line) initially with the pTet-DualOff vector (Supplementary Figure 1A) for expression of the tet-controlled transactivator (tTA) and used flow cytometry to sort the cells based on ZsGreen expression to generate a stable cell line. We then transfected this cell line with the pTRE-Dual2 vector (Supplementary Figure 1A), which contains the tet-responsive promoter (TRE) and reporter gene mCherry. We sorted for double positive cells expressing both ZsGreen and mCherry, and then analyzed the expression of the mCherry reporter protein in response to culture with and without doxycycline. Our preliminary experiment demonstrated tight, reversible gene regulation with this dual-color system (Supplementary Figure 1B).

**Figure 2:**
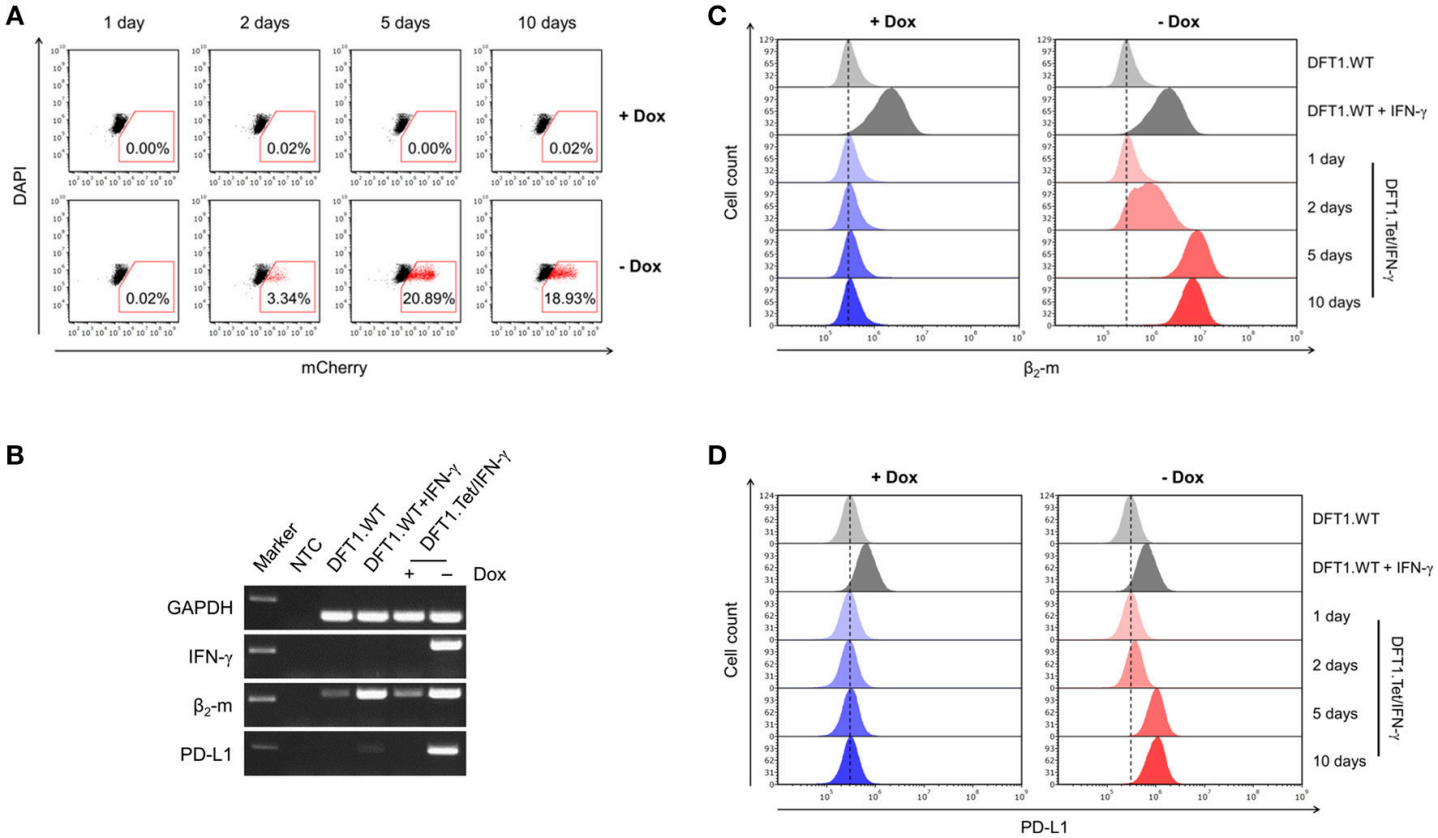
Gene expression following doxycycline removal and the downstream effects of IFN-γ. **(A)** Flow cytometric analysis of mCherry expression in DFT1.Tet/IFN-γ with and without doxycycline. DFT1.WT cells treated with and without 5 ng/ml IFN-γ for 24 h **(B,C)** or 72 h **(D)** were used as positive and negative controls for β_2_-m and PD-L1 upregulation, respectively. **(B)** mRNA expression of IFN-γ, β_2_-m and PD-L1 analyzed by RT-PCR. Results are shown for DFT1.Tet/IFN-γ cells cultured with and without doxycycline for 5 days, and GAPDH was used as a reference gene. *NTC*, no template control. Marker shows 250 bp-size band. **(C)** β_2_-m upregulation on DFT1.Tet/IFN-γ following doxycycline removal. Cells were stained with mouse anti-devil β_2_-m antibody followed by goat anti-mouse IgG conjugated to AlexaFluor 488 and were analyzed by flow cytometry. **(D)** PD-L1 upregulation on DFT1.Tet/IFN-γ following doxycycline removal. Cells were stained with mouse anti-devil PD-L1 antibody conjugated to DyLight 650 and were analyzed by flow cytometry. For both flow cytometric analyses, antibody staining of cells was performed in triplicate and dead cells were excluded by DAPI staining. The results shown are representative of *n* = 3 replicates/treatment.

We then constructed an all-in-one SB Tet-Off vector (pAF107) that contained the tet-controlled transactivator (tTA) and the tet-responsive promoter (TCE) for inducible expression of IFN-γ (Figure 1A) in DFT1 C5065 cells (DFT1.Tet/IFN-γ). The single vector approach simplifies cell line development and ensures a 1:1 ratio of transactivator and response element integration into the host cell genome. To analyze the regulation of IFN-γ by doxycycline, we split DFT1.Tet/IFN-γ into four groups of increasing periods of induction (1, 2, 5, and 10 days without doxycycline) (Figure 1B). For each group of cells with doxycycline removed, there was a replicate with doxycycline continually added. Expression of the reporter protein mCherry was quantified by flow cytometry and was used as a proxy for IFN-γ induction (Figure 2A). mCherry expression was consistently absent in all groups cultured with doxycycline, but mCherry expression was detectable by day 2 post-doxycycline removal and increased over time. These results suggest that the all-in-one system tightly regulates expression of genes downstream to the tetracycline-responsive promoter.

### MHC-I and PD-L1 Upregulation Following IFN-γ Induction

Two of the downstream effects of IFN-γ include upregulation of MHC-I and the inhibitory checkpoint molecule PD-L1 (6, 18). We used RT-PCR to initially examine mRNA levels of IFN-γ, MHC-I subunit β_2_-m, and PD-L1 in cells with and without doxycycline for 5 days to qualitatively determine expression levels before embarking on protein-based immunology. β_2_-m and PD-L1 were upregulated in cells with doxycycline removed (Figure 2B). This suggests that DFT1.Tet/IFN-γ cells produce functional IFN-γ in the absence of doxycycline. Conversely, IFN-γ, β_2_-m, and PD-L1 mRNA expression in DFT1.Tet/IFN-γ cells cultured with doxycycline was on par with expression in wild type cells.

Cell surface MHC-I and PD-L1 protein upregulation were also analyzed by flow cytometry. Surface expression of MHC-I was determined using a monoclonal antibody to β_2_-m (10). Upon doxycycline removal, β_2_-m upregulation was observed by the second day and was strongly upregulated in all cells at 5 and 10 days without doxycycline (Figure 2C). This was consistent with the temporal induction of IFN-γ observed in Figure 2A, as represented by reporter mCherry. PD-L1 upregulation following doxycycline removal also mirrored results previously obtained using exogenous IFN-γ treatment (18) (Figure 2D). As expected, β_2_-m and PD-L1 expression were absent on the surface of cells cultured with doxycycline.

### Bystander Effects of DFT1.Tet/IFN-γ

**Figure 3:**
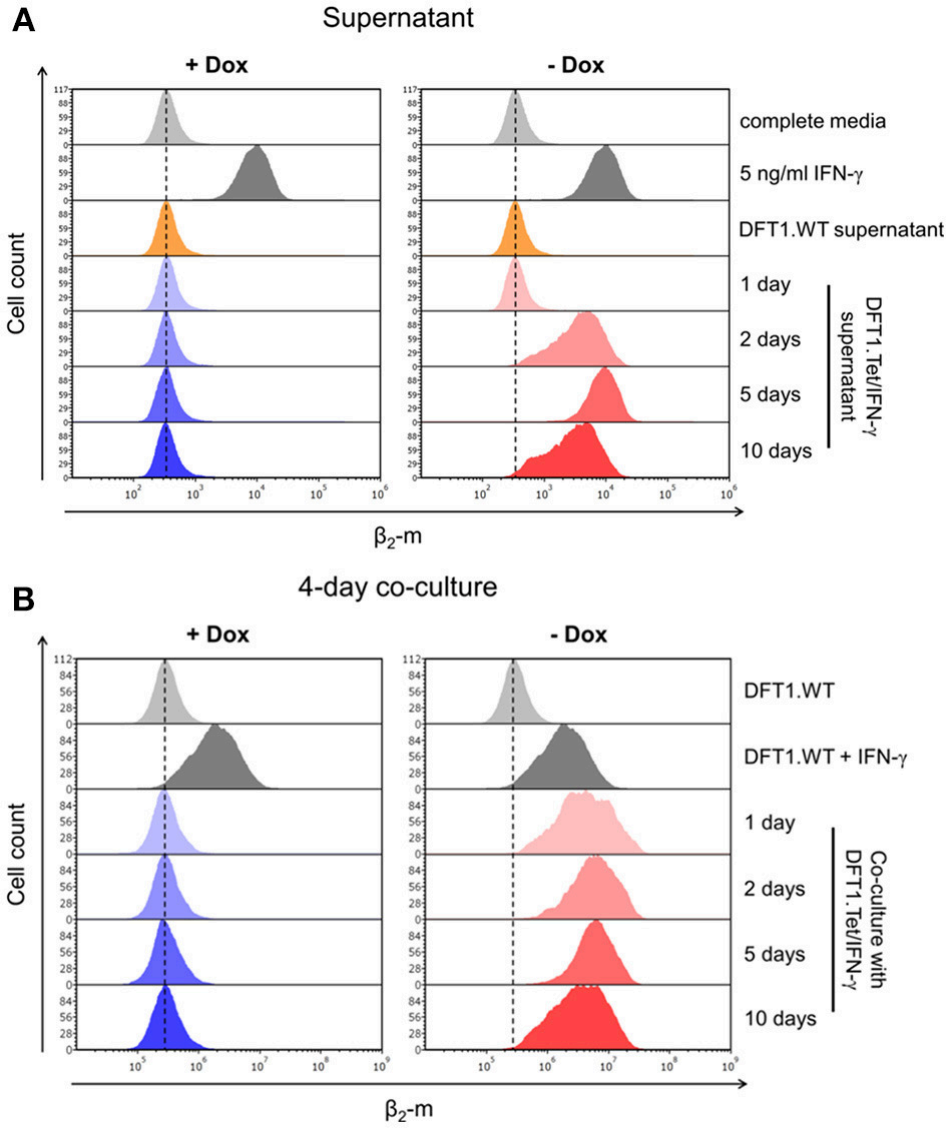
Bystander effects of DFT1.Tet/IFN-γ. **(A)** Flow cytometric analysis of β_2_-m upregulation on wild type DFT1 cells (DFT1.WT) after 48 h of stimulation with supernatant from DFT1.Tet/IFN-γ cultured with or without doxycycline for 1, 2, 5, and 10 days. Controls include DFT1.WT treated with complete media, 5 ng/ml IFN-γ or supernatant from DFT1.WT cells. **(B)** β_2_-m expression on DFT1.WT cells following 4 days of co-culture of DFT1.WT and DFT1.Tet/IFN-γ. DFT1.Tet/IFN-γ were cultured either with doxycycline (+Dox) or without doxycycline (–Dox) for 1, 2, 5, and 10 days prior to initiating the co-culture assay. DFT1.WT cells were pre-labeled with CellTrace Far Red (CTFR). The cells were stained for β_2_-m and analyzed by flow cytometry. DFT1.WT cells treated with and without 5 ng/ml IFN-γ for 24 h were used as positive and negative controls, respectively. Each treatment and co-culture were carried out in triplicate. The results shown are representative of *n* = 3 replicates/treatment.

As cytokines act on cells in both an autocrine and paracrine fashion, we wanted to determine if IFN-γ secreted by DFT1.Tet/IFN-γ could upregulate MHC-I on wild type DFT1 cells (DFT1.WT) through paracrine signaling. We collected supernatant from each group of DFT1.Tet/IFN-γ (i.e., with and without doxycycline for 1, 2, 5, and 10 days) and used the supernatant to stimulate DFT1.WT cells (Figure 1B). Consistent with the results above, β_2_-m upregulation was only observed in DFT1.WT cells treated with supernatant from DFT1.Tet/IFN-γ with doxycycline removed for 2, 5, and 10 days (Figure 3A). Due to a reduced cell number of DFT1.Tet/IFN-γ cells with doxycycline removed for 10 days (5 × 10^5^ cells as opposed to 2 × 10^6^ cells in all other groups), β_2_-m upregulation in DFT1.WT cells treated with supernatant from this group appeared less than expected. The lower number of cells for this group was a result of reduced proliferation after the last passage.

The bystander effects of IFN-γ were also investigated by co-culture of DFT1.Tet/IFN-γ with DFT1.WT cells. DFT1.Tet/IFN-γ with doxycycline removed for 1, 2, 5, and 10 days were co-cultured with DFT1.WT pre-labeled with CellTrace Far Red (CTFR), respectively, at a ratio of 1:1 for 4 days (Figure 1B). Flow cytometric analysis of β_2_-m on CTFR^+^ DFT1.WT revealed the ability of DFT1.Tet/IFN-γ cultured without doxycycline to stimulate β_2_-m upregulation on neighboring DFT1.WT cells (Figure 3B).

### Inhibition of Anti-proliferative and Pro-apoptotic Effects of IFN-γ Using Doxycycline

**Figure 4:**
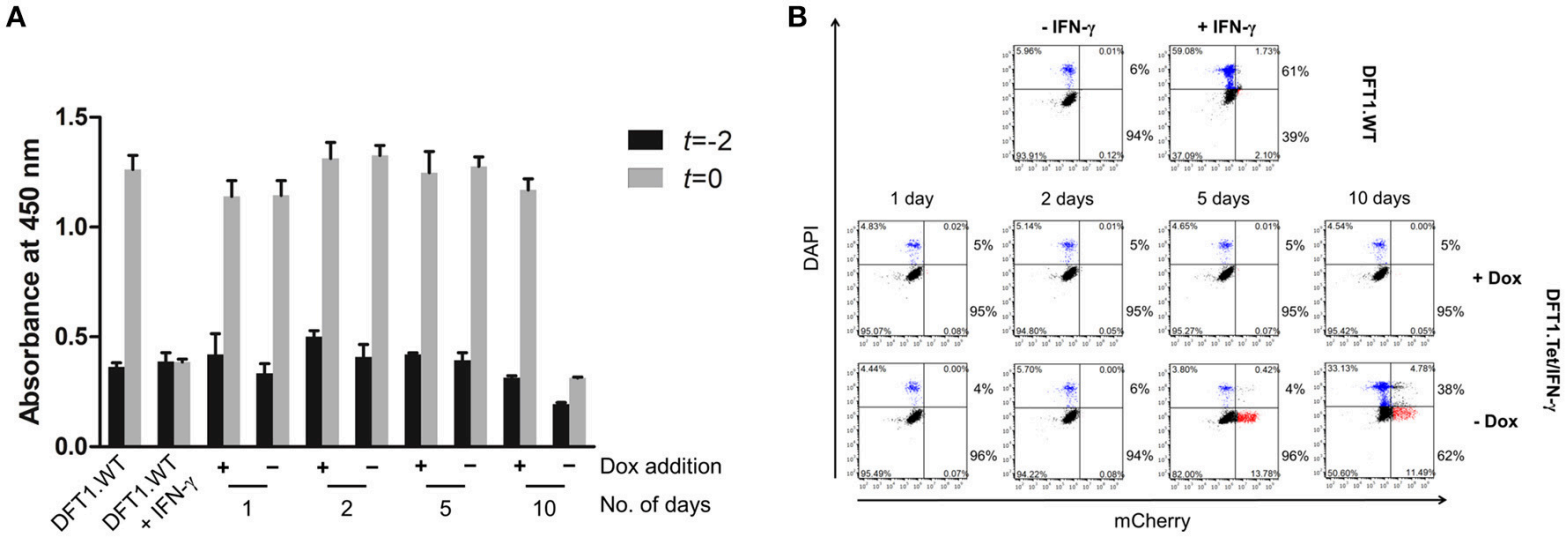
Anti-proliferative and pro-apoptotic effects of IFN-γ are ameliorated in DFT1.Tet/IFN-γ cultured with doxycycline. Cell proliferation and viability of DFT1.Tet/IFN-γ was compared against DFT1.WT cells cultured with or without 50 ng/ml IFN-γ for 10 days. **(A)** Differences in proliferation of DFT1.Tet/IFN-γ in the presence and absence of doxycycline for 1, 2, 5, and 10 days, assessed by WST-8 proliferation assay. The WST-8 assay uses absorbance at 450 nm as a proxy for the number of viable cells. Baseline absorbance was measured 2 days prior (*t* = −2) and change in absorbance was measured after 2 days (*t* = 0). Graph shows the average value of triplicates and a standard deviation error bar. **(B)** Cell viability of DFT1.Tet/IFN-γ in the presence and absence of doxycycline for 1, 2, 5, and 10 days. Cell death over 2 days (from *t* = −2 to *t* = 0) was quantitated by staining dead cells with DAPI and analysis by flow cytometry. The results shown are representative of *n* = 3 replicates/treatment. The percentages in the upper right and left quadrants were added to yield the total percentage of dead cells while the same was done for the lower quadrants to give the total percentage of viable cells.

To explore the effects of IFN-γ on cell proliferation and viability using the Tet-Off system, we examined differences in proliferation and cell death of DFT1.Tet/IFN-γ cells with and without doxycycline relative to DFT1.WT cells. Proliferation and cell death over 2 days (*t* = −2 to *t* = 0, Figure 1B) were assessed by WST-8 proliferation assay, and quantification of dead cells by DAPI staining and analysis by flow cytometry. In the absence of doxycycline, proliferation, and viability of DFT1.Tet/IFN-γ cells were reduced at 10 days, but not in cells with doxycycline removed for 1, 2, and 5 days (Figures 4A,B). DFT1.Tet/IFN-γ cells cultured with doxycycline were demonstrated to proliferate and have viabilities similar to DFT1.WT cells (Figures 4A,B).

## Discussion

Upregulation of MHC-I on DFT cells by recombinant IFN-γ prior to vaccination and immunotherapy has been associated with anti-DFT immune responses (12). Importantly, tumor regressions have been induced only when using live-MHC-I^+^ DFT cell immunotherapy in infected devils that were vaccinated prior to infection with DFT cells (11). Live-cell immunotherapy has the obvious risk of seeding new tumors into infected devils, and a live-cell prophylactic vaccine introduces the risk of seeding tumors into previously tumor-free devils. The tight regulation of IFN-γ production by the Tet-Off system allowed us to develop a cell line that can be easily cultured *in vitro* but rapidly decreases in viability and increases in immunogenicity (i.e., MHC-I upregulation) upon removal of doxycycline. Increased immunogenicity and reduced capacity for proliferation are key attributes of successful live-attenuated vaccines (32–34).

The previously used immunotherapy method of treating wild type DFT cells *in vitro* with IFN-γ prior to injecting the live MHC-I^+^ DFT cells into infected devils relies on igniting a sustained immune response for ongoing IFN-γ production. As upregulation of MHC-I is transient on DFT cells following IFN-γ exposure (28), it is possible that the pre-treated DFT cells could survive long enough *in vivo* to downregulate MHC-I expression and escape anti-tumor immunity. Although not tested *in vivo* yet due to the limitations of working with endangered species, injecting live DFT1.Tet/IFN-γ directly into facial tumors could provide elevated levels of IFN-γ production *in vivo*. This may enhance T cell-mediated anti-tumor responses, and the anti-proliferative and pro-apoptotic effects of IFN-γ on DFT cells should also increase the safety of using live tumor cells for immunotherapy. Importantly, the bystander effect of MHC-I upregulation on nearby wild type DFT cells demonstrated here could initiate a more reliable and sustained anti-tumor response.

The bystander effect of DFT1.Tet/IFN-γ shown here is also comparable to the effects of autocrine IFN-γ acting on the cells, which reinforces the use of these cells for immunotherapy. The extent of MHC-I upregulation on wild type DFT cells stimulated with supernatant from DFT1.Tet/IFN-γ without doxycycline (Figure 3A) were similar to their respective groups of DFT1.Tet/IFN-γ cells (Figure 2C) except for day 10 without doxycycline. The reduced upregulation on DFT cells treated with supernatant from this group was anticipated due to the lower number of DFT1.Tet/IFN-γ cells present compared to the other groups. This is attributed to the anti-proliferative and pro-apoptotic effects of IFN-γ with longer periods of induction. At 10 days without doxycycline, DFT1.Tet/IFN-γ had a substantially lower metabolic activity (Figure 4A), which validates the reduced proliferation observed after the last passage. Additionally, the IFNγ-mediated increased cell death shown in Figure 4B is another contributing factor to reduced cell expansion in culture.

The upregulation of PD-L1 following IFN-γ expression could pose a challenge in eliciting an effective vaccine-induced immune response. PD-L1 expression in the tumor microenvironment has been shown to inhibit T cell responses via several mechanisms such as: (i) promoting T cell apoptosis (35); (ii) inhibiting T cell activation in terms of proliferation and cytokine production (36, 37); and (iii) suppressing cytotoxic T cell killing (38). We have previously shown that PD-L1 is typically absent or expressed at very low levels on DFT cells but is upregulated by IFN-γ (18). This problem could be eliminated by knocking out the PD-L1 gene in live-attenuated DFT cells, but the bystander effect in the tumor microenvironment could lead to PD-L1-mediated inhibition of anti-tumor immunity. Alternatively, given the success of PD-1/PD-L1 blocking antibodies in human cancer, incorporating existing devil PD-1/PD-L1 blocking antibodies in immunotherapies and vaccines could be an effective means to amplify anti-DFT responses. Another potential approach that can be explored with the system developed here is to modify the tumor cells to produce the blocking antibodies (39). This would actually be more cost-effective than using purified antibodies.

The ability to suppress the anti-proliferative and pro-apoptotic effects of IFN-γ suggests that other genes that can kill tumor cells could be suppressed during cell culture but are activated during vaccination or immunotherapy. The ability to manipulate gene expression exogenously opens new avenues for DFTD vaccine research, such as incorporating “suicide genes” into DFT cells for attenuation of a live-attenuated tumor cell vaccine. Pro-apoptotic molecules such as BAX could be suppressed in culture to allow expansion of the cells, but then activated *in vivo* to ensure that new tumors are not seeded into healthy devils.

The SB transposon system provides stable integration of DNA cargo into the target cell genome (30). The stability and safety of the SB system have been demonstrated in chimeric antigen receptor T cells (CAR-T) in human clinical trials for advanced non-Hodgkin lymphoma and acute lymphoblastic leukemia (40). The simple all-in-one SB vector developed here contains both elements of the Tet-Off system, the tetracycline (tet)-controlled transactivator (tTA), and the tet-responsive promoter (TCE). Consequently, the process of generating stable transgenic cell lines by eliminating the need for dual transfection and multiple rounds of selection by cell sorting is streamlined. The ability to precisely regulate gene expression and assess downstream biological effects on tumor cells and interactions with immune cells should facilitate rapid advances in the understanding of the devil facial tumor biology and the devil immune system.

In summary, here we demonstrate the development of an effective inducible gene expression system for DFT cells using the Tet-Off system. Through this system, we were able to generate IFNγ-expressing DFT cells for MHC-I upregulation without compromising cell proliferation and viability in culture by controlling IFN-γ expression at a transcriptional level. The ability to precisely manipulate gene expression using the Tet-Off system has also allowed us to establish a method of studying gene function in Tasmanian devils and is the first step toward a live-attenuated DFT vaccine.

## Author Contributions

CO performed all lab experiments, analyzed data, prepared the figures, and wrote the manuscript. AF provided intellectual input and contributed to writing and revision of the manuscript. GW and AL provided intellectual input and revised the manuscript.

### Conflict of Interest Statement

The authors declare that the research was conducted in the absence of any commercial or financial relationships that could be construed as a potential conflict of interest.

## Abbreviations

DFTD: devil facial tumor disease
DFT1: devil facial tumor 1
DFT2: devil facial tumor 2
tet: tetracycline
dox: doxycycline
tTA: tet-controlled transactivator
TCE: tet-responsive promoter
SB: Sleeping Beauty.
