# Supplementary material for: Inducible IFN-γ Expression for MHC-I Upregulation in Devil Facial Tumor Cells
Source: Front Immunol. 2019 Jan 14;9:3117. doi: 10.3389/fimmu.2018.03117 (PMC6340284; doi:10.3389/fimmu.2018.03117)
Supplement: Supplementary file 1 [file Data_Sheet_1.docx]

Supplementary Material

**Inducible IFN-γ Expression for MHC-I Upregulation in Devil Facial Tumor Cells**

**Chrissie E. B. Ong^*^, A. Bruce Lyons, Gregory M. Woods, Andrew S. Flies^*^**

*** Correspondence:** Chrissie E. B. Ong: [chrissie.ong@utas.edu.au](mailto:chrissie.ong@utas.edu.au),

Andrew S. Flies: [andy.flies@utas.edu.au](mailto:andy.flies@utas.edu.au)

**Supplementary Figure 1.** The Tet-Off system in DFT1 cell line C5065

**Supplementary Table 1.** Primers and PCR cycling conditions for construction of pAF107

**Supplementary Table 2.** Primers and PCR cycling condition for RT-PCR of GAPDH, IFN-γ, β_2_-m and PD-L1


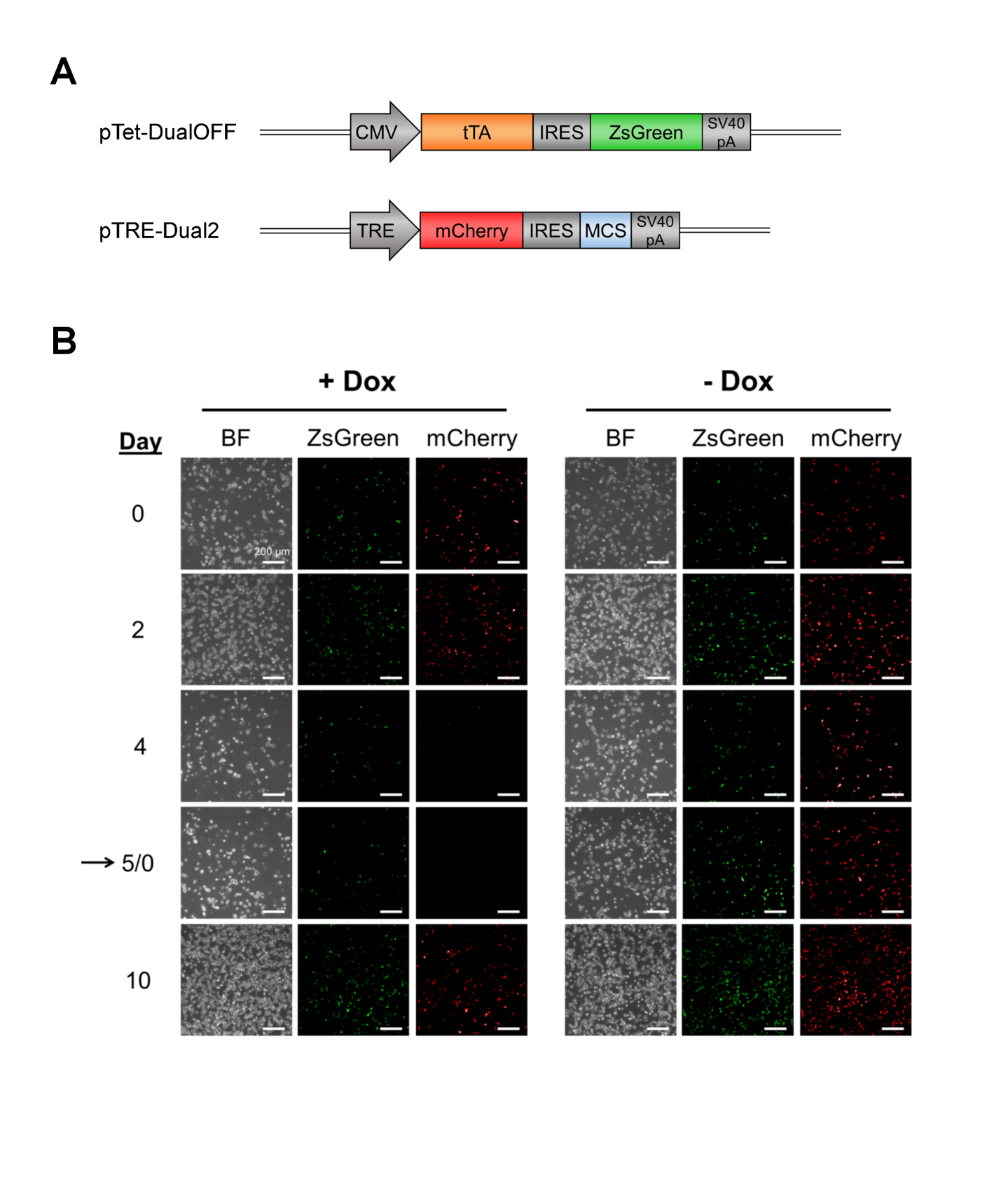
**Supplementary Figure 1.** The Tet-Off system in DFT1 cell line C5065. **(A)** Expression vectors from Clontech: pTet-DualOFF and pTRE-Dual2 for introduction of the Tet-Off system in DFT1 cells. *tTA*, tetracycline (tet)-controlled transactivator; *TRE*, tet-responsive promoter; *MCS*, multiple cloning site. **(B)** Gene expression was suppressed or induced by adding or removing doxycycline to the cell culture. C5065 cells transfected with pTet-DualOFF and pTRE-Dual2, and cultured in the absence of doxycycline were subjected to gene suppression by adding 100 ng/ml doxycycline and were assessed for changes in expression of tet-controlled reporter mCherry. ZsGreen is constitutively expressed and serves as a reporter for expression of tTA transactivator. Once complete gene suppression was achieved (i.e. at day 5 after doxycycline addition), doxycycline was removed (as shown by arrow) to demonstrate reversibility of doxycycline inhibition. The results show the suppression of tet-controlled gene mCherry following doxycycline addition, achieving complete suppression after 5 days. Once doxycycline was removed from the culture medium, gene expression was fully restored after 10 days.

**Supplementary Table 1.** Primers and PCR cycling conditions for construction of pAF107

| **Vector** | **Template DNA** | **Target DNA** | **Product size (bp)** | **Primers** | **Primer sequence (5' to 3')** | **Reaction conditions** |
| --- | --- | --- | --- | --- | --- | --- |
| pAF67 | pTRE-Dual2 | Linearized vector | 3840 | pTRE-Dual2-XbaI-linear.FOR | AGGATCATAATCAGCCATACCACATTTGT  AGAG | 1x: 95°C for 3 min;  35x: 98°C for 15 s, 63°C for 15 s, 72°C for 3 min;  1x: 72°C for 5 min |
|  |  |  |  | pTRE-Dual2-NotI-linear.REV | GCGGTTGTGGCCATATTATCATCGTG |  |
|  | pAF23 | Devil IFN-γ cDNA | 545 | pAF67.FOR | GATAATATGGCCACAACCGCCATGAATTA  TTCAAGCTACCTCTTAGC | 1x: 95°C for 3 min;  10x: 98°C for 15 s, 65 to 55°C (-1 °C each cycle) for 15 s, 72°C for 30 s;  25x: 98°C for 15 s, 65°C for 15 s, 72°C for 30 s;  1x: 72°C for 5 min |
|  |  |  |  | pAF67.REV | GTATGGCTGATTATGATCCTCTACTGTGTG  ATTTTTCCTTGGC |  |
| pAF107 | pSBtet-RH | Linearized vector | 3329 | pAF107_vec1.FOR | ACTGTGATCAATTAGTTCGAAGGCCTGTC  GTGAAGC | 1x: 95°C for 3 min;  10x: 98°C for 15 s, 70 to 60°C (-1 C each cycle) for 15 s, 72°C for 3 min;  25x: 98°C for 15 s, 65°C for 15 s, 72°C for 3 min;  1x: 72°C for 5 min |
|  |  |  |  | pAF107_vec1.REV | GCCCTTGCTCACCATGGTGGCCTCAGAGG |  |
|  | pAF67 | Fragment 1 | 1841 | pAF107_frag1.FOR | CCTCTGAGGCCACCATGGTGAGCAAGGGC |  |
|  |  |  |  | pAF107_frag1.REV | TTGGCCTGACAGGCCTACTGTGTGATTTTT  CCTTGGCTTTTGTTC |  |
|  | pSBtet-RH | Fragment 2 | 909 | pAF107_frag2.FOR | GAAAAATCACACAGTAGGCCTGTCAGGCC  AAGC |  |
|  |  |  |  | pAF107_frag2.REV | GTCCAGTCTAGACATGGTGGCCTCAGGTGC |  |
|  | pTet-DualOFF | Fragment 3 | 775 | pAF107_frag3.FOR | GCACCTGAGGCCACCATGTCTAGACTGGA  CAAGAGCAAAGTC |  |
|  |  |  |  | pAF107_frag3.REV | CGACGTCACCAGCCTGCTTCAGCAGGCTG  AAGTTAGTAGCTCCACTGCCCCCGGGGAG  CATGTCAAGGTCC |  |
|  | pAF56.1 | Fragment 4 | 783 | pAF107_frag4.FOR | CAGCCTGCTGAAGCAGGCTGGTGACGTCG  AGGAGAATCCTGGCCCCATGGGCCCTTCG  GACCCAG |  |
|  |  |  |  | pAF107_frag4.REV | GCTTCACGACAGGCCTTCGAACTAATTGA  TCACAGTTAATGTCCCAGAATCG |  |
|  | Fusion of fragments 1, 2, 3 and 4 | Fragment 1-4 | 4222 | pAF107_frag1.FOR | CCTCTGAGGCCACCATGGTGAGCAAGGGC | 1x: 95°C for 3 min;  30x: 98°C for 15 s, 65°C for 15 s, 72°C for 5 min;  1x: 72°C for 5 min |
|  |  |  |  | pAF107_frag4.REV | GCTTCACGACAGGCCTTCGAACTAATTGA  TCACAGTTAATGTCCCAGAATCG |  |

**Supplementary Table 2.** Primers and PCR cycling condition for RT-PCR of devil GAPDH, IFN-γ, β_2_-m and PD-L1

| **Target** | **Primers** | **Primer sequence (5' to 3')** | **Product size (bp)** | **Reaction conditions** |
| --- | --- | --- | --- | --- |
| GAPDH | cadeGAPDH_F1 | acacccactcttccacctt | 143 | 1x: 98°C for 30 s;  30x: 98°C for 10 s, 60°C for 30 s, 72°C for 1 min  1x: 72°C for 5 min |
|  | cadeGAPDH_R1 | ttactccttggaggccatgta |  |  |
| IFN-γ | deIFNg_seq.FOR | GCAAACTCTTCACAACTACTTTAATGC | 310 |  |
|  | deIFNg_seq.REV | AGCTTTCCTTTGGACTTTGAGG |  |  |
| β_2_-m | B2Mex1F | ATGGTCACAAGTCCTCCCAGAGTTC | 301 |  |
|  | B2Mex2R | GCACCAAGTTCTGTTCTGGATCCCATTTAATTAC |  |  |
| PD-L1 | deB7H1_318_F | AGATGCTGGGGCTTACCGCTGTATT | 280 |  |
|  | deB7H1_597_R | TGTGGCATTGACCCTGAGAGTGCT |  |  |
